# The Special Neuraminidase Stalk-Motif Responsible for Increased Virulence and Pathogenesis of H5N1 Influenza A Virus

**DOI:** 10.1371/journal.pone.0006277

**Published:** 2009-07-17

**Authors:** Hongbo Zhou, Zhengjun Yu, Yong Hu, Jiagang Tu, Wei Zou, Yaping Peng, Jiping Zhu, Yongtao Li, Anding Zhang, Ziniu Yu, Zhiping Ye, Huanchun Chen, Meilin Jin

**Affiliations:** 1 State Key Laboratory of Agricultural Microbiology, College of Veterinary Medicine, Huazhong Agricultural University, Wuhan, the People's Republic of China; 2 Laboratory of Pediatric and Respiratory Viral Diseases, Division of Viral Products, Office of Vaccine Research and Review, Center for Biologics Evaluation and Research, Food and Drug Administration, Bethesda, Maryland, United States of America; Institute of Infectious Disease and Molecular Medicine, South Africa

## Abstract

The variation of highly pathogenic avian influenza H5N1 virus results in gradually increased virulence in poultry, and human cases continue to accumulate. The neuraminidase (NA) stalk region of influenza virus varies considerably and may associate with its virulence. The NA stalk region of all N1 subtype influenza A viruses can be divided into six different stalk-motifs, H5N1/2004-like (NA-wt), WSN-like, H5N1/97-like, PR/8-like, H7N1/99-like and H5N1/96-like. The NA-wt is a special NA stalk-motif which was first observed in H5N1 influenza virus in 2000, with a 20-amino acid deletion in the 49^th^ to 68^th^ positions of the stalk region. Here we show that there is a gradual increase of the special NA stalk-motif in H5N1 isolates from 2000 to 2007, and notably, the special stalk-motif is observed in all 173 H5N1 human isolates from 2004 to 2007. The recombinant H5N1 virus with the special stalk-motif possesses the highest virulence and pathogenicity in chicken and mice, while the recombinant viruses with the other stalk-motifs display attenuated phenotype. This indicates that the special stalk-motif has contributed to the high virulence and pathogenicity of H5N1 isolates since 2000. The gradually increasing emergence of the special NA stalk-motif in H5N1 isolates, especially in human isolates, deserves attention by all.

## Introduction

The neuraminidase (NA) of influenza A viruses, a type II membrane glycoprotein, is one of two major glycoproteins on the virus surface. The NA plays a central role in the release of the virus from infected cells by removing terminal sialic acids from oligosaccharide side chains to which the viral haemagglutinin (HA) binds. The NA protein is a tetramer with a boxlike head comprised of four roughly spherical subunits, as well as centrally attached stalk with a hydrophobic region by which the stalk is embedded in the viral membrane. The NA stalk region varies considerably among different viruses, even within the same subtypes. The distinct antigenic properties of different HA and NA molecules are used to classify influenza type A viruses into subtypes: 16 for HA (H1–H16) and 9 for NA (N1–N9). The NA sequence analysis of all N1 subtype influenza A viruses in GenBank showed that the NA stalk region could be divided into six different stalk-motifs, A/Gs/Gd/1/96/H5N1-like (with no amino acid deletion in the NA stalk region), A/WSN/33/H1N1-like (16 residues deleted between amino acids 57 and 72), A/Puerto Rico/8/34/H1N1-like (15 residues deleted between amino acids 63 and 77), A/Hong Kong/156/97/H5N1-like (19 residues deleted between amino acids 54 and 72), A/Chicken/Italy/1067/99/H7N1-like (22 residues deleted between amino acids 54 and 75) and A/chicken/Hubei/327/2004/H5N1-like (20 residues deleted between amino acids 49 and 68) [1]. A previous study showed that a spontaneous NA mutant of influenza A virus, characterized by an 18-amino-acid deletion in the stalk, lacked enzyme activity with a large substrate (fetuin) but not with a small substrate (sialyllactose) and could not release viruses bound to erythrocytes [2]. Castrucci et al. [3] and Luo et al. [4] investigated the biologic importance of the NA stalk by generating WSN viruses with a deletion, insertion or mutation of the NA stalk region. The results showed that the length of the NA stalk could be variable and correlated with replication and pathogenesis of influenza virus.

Since 1997, the amino acid deletion in the NA stalk also had been found in H5N1 influenza virus. Since 2000, a new special NA stalk-motif had been observed in H5N1 influenza virus, with a 20-amino acid deletion in the 49^th^ to 68^th^ in the stalk region [5]–[10], which was different from previous H5N1 strains isolated from 1996 to 1999 with a 19-amino acid deletion in the 54^th^ to 72^nd^ or with no amino acid deletion in the NA stalk region. There was a gradually increasing percentage of the special NA stalk-motif in all H5N1 isolates from 2000 (15.8%) to 2007 (100%). However, the biologic characteristic of NA stalk-motif and whether NAs with different stalk-motifs caused the difference of virulence and pathogenesis in H5N1 influenza virus had not yet been studied in detail.

Here we reported the application of reverse genetics in assessing the biologic importance of the different NA stalk-motif in H5N1 influenza A viruses. Our findings indicated that the NA stalk-motif played a critical role in virulence and pathogenesis of H5N1 avian influenza virus and the special NA stalk-motif (a 20-amino acid deletion in the 49^th^ to 68^th^ in the stalk region) may be an important one among the reasons contributing to the emergence of H5N1 isolates with increased virulence since 2000.

## Materials and Methods

### Sequence alignment comparison of the NA stalk region of H5N1 influenza A virus

Published NA sequences of 1411 H5N1 influenza A viruses used for comparison were obtained from 1996 to 2007 from the Influenza Virus Resource (http://www.ncbi.nlm.nih.gov/genomes/FLU/FLU.html). These NA sequences were aligned and the stalk regions were compared.

### Viruses and plasmids

**Figure 1:**
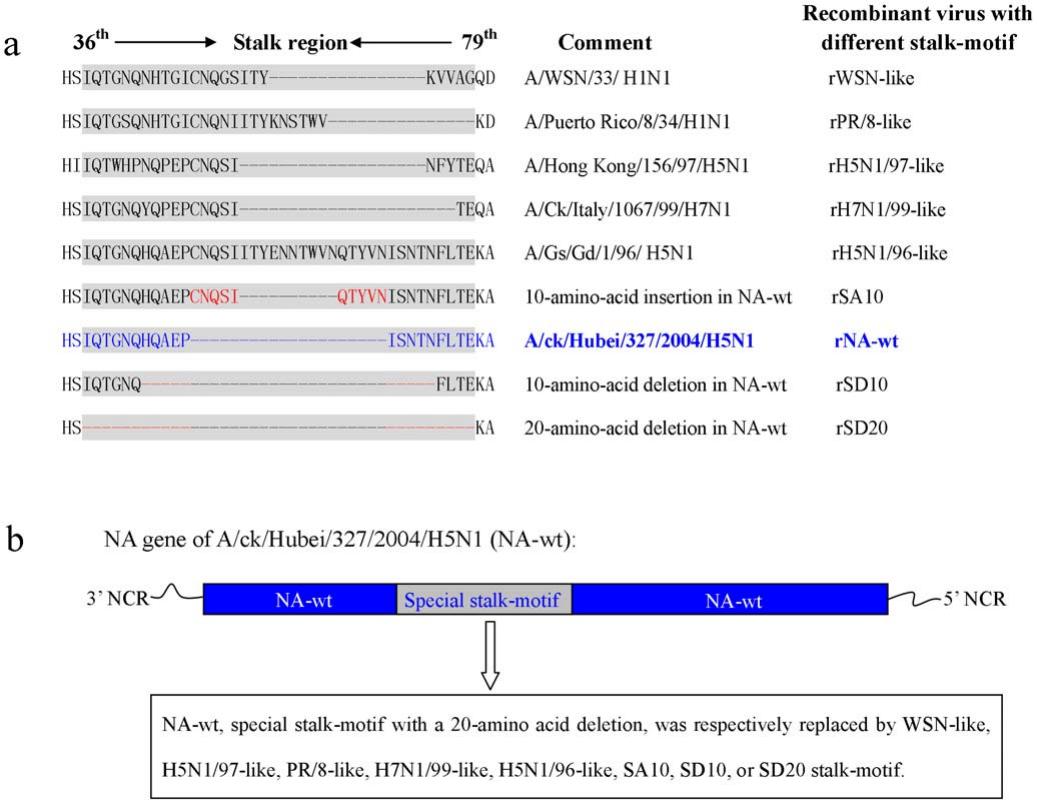
Different NA stalk-motifs and NA mutants. (a) Alignment comparisons of deduced amino acid sequences of the NA stalk region. Representative sequences of N1 subtype influenza A viruses collected from 1918 to 2004 were compared. The NA sequence analysis of all N1 subtype influenza A viruses in GenBank showed that the NA stalk region could be divided into six different stalk-motifs, WSN-like, PR/8-like, H5N1/97-like, H7N1/99-like, H5N1/96-like and NA-wt. The dash means the amino acid was deleted at that site. The deleted and inserted amino acids in NA-wt were indicated with red color. (b) The stalk region of NA gene of A/chicken/Hubei/327/2004/H5N1 was replaced by different stalk motifs and nine different stalk-motif NAs were obtained to generate different stalk-motif recombinant viruses.

Plasmids pHW-181-PB2, pHW-182-PB1, pHW-183-PA, pHW-185-NP, pHW-187-M and pHW-188-NS contain the corresponding genes of A/WSN/33 (H1N1). The H5N1 viruses A/chicken/Hubei/327/2004 (CKDW/04) was isolated in central China during the outbreak of the highly pathogenic H5N1 avian influenza virus (AIV) in the spring of 2004. Pathogenesis tests in BALB/c mice and SPF chickens indicated that CKDW/04 was highly pathogenic to mice and had a chicken intraveneous pathogenesis index (IVPI) of 3.0 [11]. The sequence accession numbers of HA and NA genes from CKDW/04 are AY684706 and AY684708, respectively. Two plasmids pHW-HA and pHW-NA-wt with 20 amino acid residue deleted NA (special NA stalk-motif) containing HA and NA genes of CKDW/04 respectively, and other plasmids used to rescue NA stalk mutants, pHW-NA-WSN-like, pHW-NA-PR/8-like, pHW-NA-H5N1/97-like, pHW-NA-H7N1/99-like, pHW-NA-H5N1/96-like, pHW-NA-SA10, pHW-NA-SD10 and pHW-NA-SD20 are derivatives of pHW-NA-wt by either deleting or inserting nucleotides in the region encoding the NA stalk, using oligonucleotide-directed mutagenesis. The recombinant viruses contain different NA stalk-motifs with the stalk-motif of wild-type NA replaced with the other different stalk-motifs (Fig. 1). All works with live H5N1 viruses were conducted in the ABSL-3 facilities at Huazhong Agricultural University.

### Generation of 7∶1 recombinant viruses by reverse genetics

Nine recombinant H5N1 viruses, rNA-wt, rWSN-like, rH5N1/97-like, rPR/8-like, rH7N1/99-like, rH5N1/96-like, rSA10, rSD10 and rSD20 were generated by 8-plasmid based reverse genetics system as described previously [12], [13]. Nine plasmids containing the NA gene with different stalk-motifs derived from NA of CKDW/04 (H5N1) were used separately with seven plasmids containing PB2, PB1, PA, NP, M, NS gene from A/WSN/33 (H1N1) and HA from CKDW/04 (H5N1) respectively.

### Virus plaque assay

As described previously [14], for plaque assays, confluent MDCK cells in six-well plates were inoculated with appropriate dilutions of virus in DMEM containing 1% BSA for 1 h at 37°C. Unbound virus was removed by washing with PBS. Cells were then overlaid with DMEM (without phenol red, HyClone)-0.8% agarose mixture and incubated at 37°C. After 3 days, a second agar overlay containing 1∶10,000 neutral red was added to facilitate plaque counting.

### Virus elution assay

The ability of the NA to elute virus bound on erythrocytes was assessed as described previously [3]. Briefly, fifty microliters of twofold dilutions of virus containing the HA titers of 1∶128 was incubated with 50 µl of 0.5% chicken erythrocytes in microtiter plates at 4°C for 1 h. The microtiter plates were then stored at 37°C, and the reduction of HA titers was recorded periodically for 10 h. Calcium saline (6.8 mM CaCl2-154 mM NaCl in 20 mM borate buffer, pH 7.2) was used as a diluent.

### MDT and EID_50_ in chicken embryos

The virulences of the nine recombinant viruses were determined by the mean death time (MDT) and 50% egg infectious dose (EID_50_) in embryonated specific-pathogen-free (SPF) chicken eggs. The MDT recorded was the mean time (in hours) required for the minimum lethal dose to kill the embryos and the EID_50_ was calculated by the method of Reed and Muench.

### Pathogenesis studies in chickens

Groups of 10 6-week-old SPF White Leghorn chickens were used in the chicken IVPI test with the recombinant viruses. Briefly, chickens were inoculated intravenously with 0.2 ml of a 1∶10 dilution of allantoic fluid containing the recombinant viruses and were observed clinically for 10 days and IVPI was determined by OIE (Office International Des Epizooties) standard procedure. Additional pathogenesis tests were also conducted in chickens for mortality and virus shedding tests. Groups of 10 6-week-old SPF chickens were inoculated intravenously with the recombinant viruses respectively at a dose of 10^6^ EID_50_. Mortality was observed for 10 days and viruses in the cloacal swabs on day 3 p.i. were titrated by plaque assay in MDCK cells. All the experimental protocols were approved by the Laboratory Animal Monitoring Committee of Hubei province of China and performed accordingly.

### Pathogenesis studies in mice

Six-week-old female BALB/c mice, were anesthetized with methoxyflurane and 50 µl of infectious virus diluted in PBS was inoculated intranasally (i.n.). The MLD_50_ titers were determined by inoculating groups of four mice i.n. with serial 10-fold dilutions of virus, and were calculated by the method of Reed and Muench and were expressed as the log10 EID_50_ required to give 1MLD_50_. For comparison of morbidity (measured by weight loss), mortality, and virus distribution in different tissues, additional mice were infected with inoculating doses of 1000EID_50_ of the recombinant viruses. Mice were observed daily for 14 days for weight loss and mortality. On days 3, 6, 9 and 12 p.i., three mice from each group were sacrificed and heart, liver, spleen, lung, kidney, brain, and rectum samples were harvested. Virus titers in the tissue homogenates were determined by plaque assay in MDCK cells. The virus titer in the tissue was expressed in log10 pfu/g. The lower limit of virus detection was 2 log10 PFU per 1 g tissue. All the experimental protocols were approved by the Laboratory Animal Monitoring Committee of Hubei province of China and performed accordingly.

## Results

### Sequence Alignment Comparison of the NA Stalk Region of H5N1 Influenza A Virus

**Figure 2:**
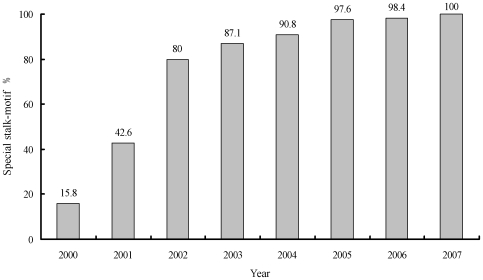
The ratio of the special stalk-motif in H5N1 isolates from 2000 to 2007. Published NA sequences of 1411 H5N1 influenza A viruses used in this study for comparison were obtained from the Influenza Virus Resource from 1996 to 2007. These NA sequences were aligned and the stalk regions were compared. The special NA stalk-motif was observed in three H5N1 strains of the total 19 strains in 2000, 29 of 68 strains in 2001, 52 of 65 strains in 2002, 88 of 101 strains in 2003, 247 of 272 strains in 2004, 368 of 377 strains in 2005, 360 of 366 strains in 2006 and 99 of 99 strains in 2007, and it was not observed in all 44 H5N1 strains from 1996 to 1999.

The special NA stalk-motif was observed in three H5N1 strains of the total 19 strains in 2000, 29 of 68 strains in 2001, 52 of 65 strains in 2002, 88 of 101 strains in 2003, 247 of 272 strains in 2004, 368 of 377 strains in 2005, 360 of 366 strains in 2006 and 99 of 99 strains in 2007 (Fig. 2). There was a ratio increase of the special NA stalk-motif in H5N1 isolates from 2000 to 2007 and it was observed in all H5N1 isolates in 2007. The distribution of the special NA stalk-motif in H5N1 mammalian isolates also gradually increased. In swine viruses, the special NA stalk-motif was observed in 1 of the total 5 strains isolated from 2001 to 2003, 3 of 4 strains in 2004. In tiger viruses, it was observed in all 11 isolates from 2004 to 2005. Notably, in human viruses, it was only observed in 1 of the total 5 strains in 2003, but it was observed in all 173 strains from 2004 to 2007. These revealed that the special NA stalk-motif may have a selective advantage in the evolution of H5N1 influenza A virus.

### The Generation and Characterization of Influenza Virus Mutants Containing Different NA Stalk-Motifs

By using the PB2, PB1, PA, NP, M and NS from A/WSN/33 and the HA and NA of a highly pathogenic avian isolated strain, A/chicken/Hubei/327/2004/H5N1, as genetic backbone, the different stalk-motifs of NAs were altered by mutagenesis, and the different strains carrying these mutated NA genes were made by reverse genetics to represent the sequences found at the NA stalk region of various N1 subtype influenza virus isolates. Nine 7∶1 recombinant H5N1 viruses were generated, rNA-wt, rWSN-like, rH5N1/97-like, rPR/8-like, rH7N1/99-like, rH5N1/96-like, rSA10, rSD10 and rSD20.

**Figure 3:**
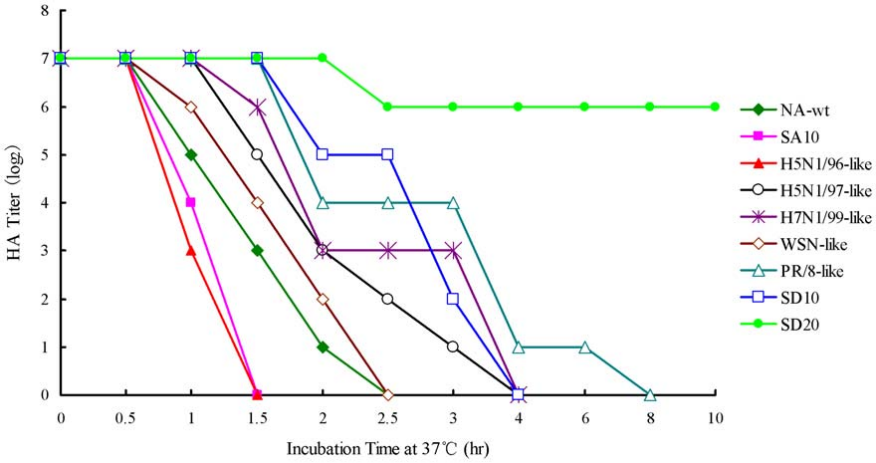
Virus elution from erythrocytes. Twofold dilutions of virus containing the HA titers of 1∶128 (2^7^) were incubated with equal volume of 0.5% chicken erythrocytes in microtiter plates at 4°C for 1 h. The microtiter plates were then stored at 37°C, and the reduction of HA titers was recorded periodically for 10 h.

We compared the ability of the mutant NAs to release erythrocyte bound virions, first by performing hemagglutination at 4°C followed by incubation of the HA microtiter plates at 37°C. If the NA was active, the bound virions would be released due to the viral NA. Comparison of the elution properties of the NA stalk mutants revealed the correlation between different stalk-motifs and the ability of the NA to release virions from erythrocytes (Fig. 3). The rH5N1/96-like and rSA10 were released completely from erythrocytes by 1.5 h of incubation at 37°C, whereas longer times were required for release of rNA-wt, rWSN-like, rH5N1/97-like and rH7N1/99-like. The rSD10 and rPR/8-like displayed a slower release from erythrocytes than did the other viruses. The rSD20 were released only slightly, even after 10 h of incubation.

As shown in Table 1, rNA-wt had an EID_50_ (50% egg infectious dose) similar to that of all other NA stalk mutants except rSD20, ranging from 8.0 to 8.4, whereas EID_50_ of rSD20 was about 8-fold lower than rNA-wt. For virus titers on chicken embryos (HA (log2)) and MDCK cells (PFU), the longer stalk viruses rSA10 and rH5N1/96-like showed slightly higher than those of the other viruses of similar virus titers. The rNA-wt had a MDT (mean death time) on chicken embryos similar to that of rWSN-like and rH5N1/97-like (36 to 38 h), whereas MDTs of rH7N1/99-like, rH5N1/96-like, rPR/8-like, rSA10 and rSD10 showed 5- to 12-hour delay. Importantly, compared with rNA-wt, rSD20 was significantly attenuated to chicken embryos and with a MDT of 55.7 hours.

**Table 1:** In vitro and in vivo characteristics of the recombinant viruses.

Virus	Study in embryonated chicken eggs			Plaque in MDCK cells		Pathogenesis studies in chickens			Study in mice
	EID_50_ (log_10_)	HA titer (log2)	MDT^a^ (hour)	PFU/ml	Size	IVPI^b^	Mortality^c^	Virus titer^d^	MLD_50_ (log_10_EID_50_)
rWSN-like	8.4	8.6	37.4	2.3×10^8^	>2 mm	1.21	4/10	3.4±0.6	1.81
rPR/8-like	8.1	8.7	45.3	2.1×10^8^	1–2 mm	0.41	1/10	2.3±0.5	2.91
rH5N1/97-like	8.4	8.7	38.2	2.1×10^8^	>2 mm	1.21	4/10	3.5±0.5	1.87
rH7N1/99-like	8.0	8.5	47.5	2.0×10^8^	1–2 mm	0.37	1/10	2.6±0.6	2.30
rH5N1/96-like	8.2	9.1	42.5	3.7×10^8^	1–2 mm	0.43	1/10	2.7±0.2	2.34
rSA10	8.1	8.8	45.4	3.1×10^8^	1–2 mm	0.46	1/10	2.5±0.5	2.08
rNA-wt	8.4	8.7	36.2	2.3×10^8^	>2 mm	1.43	4/10	3.7±0.3	1.75
rSD10	8.0	8.5	49.8	2.0×10^8^	1–2 mm	0.45	1/10	2.1±0.3	2.30
rSD20	7.5	8.1	55.7	1.1×10^8^	1–2 mm	0	0/10	-	3.50

a MDT (mean death time), average number of hours required to kill the inoculated eggs.

b According to the criteria set by OIE for evaluating the pathogenesis in chickens, the viruses with an IVPI value greater than 1.2 are defined as highly pathogenic avian influenza virus (HPAIV), or are defined as low pathogenic avian influenza virus (LPAIV).

c Number of birds that died/number of birds inoculated.

d The mean virus titer was expressed as the log10 PFU/ml±standard deviation. “-”, virus was not recovered from the cloacal swabs in inoculated chickens on day 3 p.i.

For plaquing on MDCK cells, rNA-wt, rWSN-like and rH5N1/97-like displayed larger plaque size compared with that displayed by rH7N1/99-like, rH5N1/96-like, rPR/8-like, rSA10, rSD10 and rSD20.

### Pathogenesis Studies of Recombinant Viruses in Chickens

According to the criteria set by OIE for evaluating the pathogenesis in chickens, the virus with an IVPI value greater than 1.2 was defined as highly pathogenic avian influenza virus (HPAIV). Therefore, rNA-wt, rWSN-like and rH5N1/97-like viruses belonged to HPAIV whereas rPR/8-like, rH7N1/99-like, rH5N1/96-like, rSA10 and rSD10 with the IVPI value of 0.41, 0.37, 0.43, 0.46 and 0.45, respectively, were defined as low pathogenic avian influenza virus (LPAIV) (Table 1). The rSD20 did not cause any disease signs or deaths in chicken.

Additional pathogenesis tests were also conducted in chickens. Groups of 10 4-week-old SPF chickens were inoculated intravenously with the recombinant viruses respectively at a dose of 10^6^ EID_50_. The mortality and virus shedding tests in chickens also indicated that rNA-wt, rWSN-like and rH5N1/97-like caused more deaths (4/10) and more viral shedding from cloaca than did rPR/8-like, rH7N1/99-like, rH5N1/96-like, rSA10 and rSD10 (Table 1). And rSD20 did not cause any visible disease or deaths in chicken and no virus shedding was seen.

### In Vivo Characterization of Recombinant Viruses in the Mouse Model

The MLD_50_ (50% mouse lethal dose) of the recombinant viruses were determined after inoculation of groups of four mice with 10^1^, 10^2^, 10^3^, 10^4^ and 10^5^ EID_50_. As shown in Table 1, rNA-wt had a MLD_50_ similar to that of rWSN-like and rH5N1/97-like, whereas MLD_50_ of rPR/8-like, rH7N1/99-like, rSA10 and rSD10 were respectively 14.5-fold, 3.5-fold, 2.1-fold and 3.5-fold higher than that of rNA-wt. Importantly, the MLD_50_ of rSD20 with the stalkless mutant and rH5N1/96-like (rSA20) with no deletion stalk mutant were 56.2-fold and 12.3-fold higher than that of rNA-wt, respectively.

**Figure 4:**
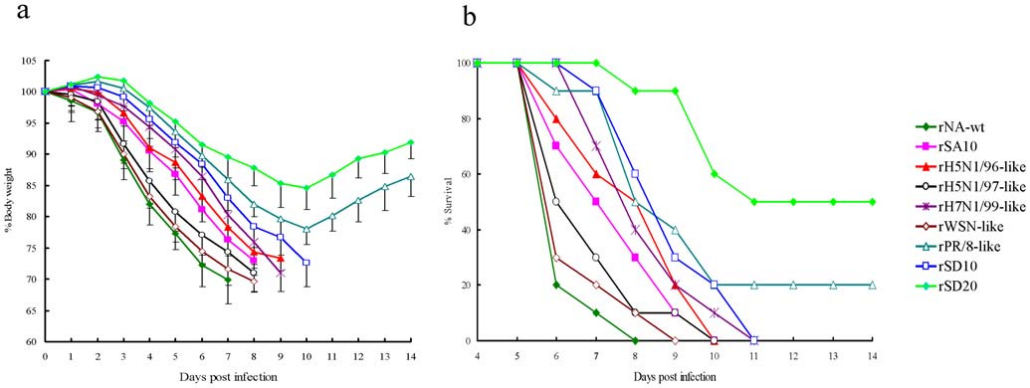
Weight loss and Mortality of mice inoculated with strains having varying NA stalk lengths. Six-week-old BALB/c mice were inoculated intranasally with 1000EID_50_ of the recombinant influenza viruses, with 10 mice per group. (a) The body weight of mice infected was measured up to 14 days p.i. (b) The data expressed as the survival percentage of mice infected with 1000EID_50_.

For comparison of morbidity (measured by weight loss) and mortality, additional mice were inoculated intranasally 1000EID_50_ of the recombinant viruses. The body weight of each mouse was measured daily until 14 days p.i. All mice infected with any of the recombinant viruses had extensive body weight loss. The rH7N1/99-like, rH5N1/96-like, rSA10 and rSD10 caused rapid weight loss. The rNA-wt, rWSN-like and rH5N1/97-like caused the most rapid body weight loss and rNA-wt resulted in the greatest weight loss (Fig. 4a). The rPR/8-like and rSD20 showed significantly lower weight loss than did rNA-wt over the 10 days p.i. (*P*<0.001).

When the data were plotted as a survival curve, all of the NA stalk mutant viruses led to increased survival compared with rNA-wt (Fig. 4b). The rNA-wt caused the most rapid death in mice which started on 5 days p.i. and all of the mice in the virus group were dead by 8 days p.i. However, mice infected with rWSN-like, rH5N1/97-like, rH7N1/99-like, rH5N1/96-like, rSA10 or rSD10 displayed 1- to 3-day delay in death compared with mice infected with rNA-wt, and all mice except rPR/8-like and rSD20 groups succumbed to infection by day 11 p.i. (Fig. 4b). In contrast to the lethal outcome in mice infected with rNA-wt, the rPR/8-like and rSD20 were significantly attenuated resulting in 20% and 50% survival by 14 days p.i., respectively (Fig. 4b).

**Figure 5:**
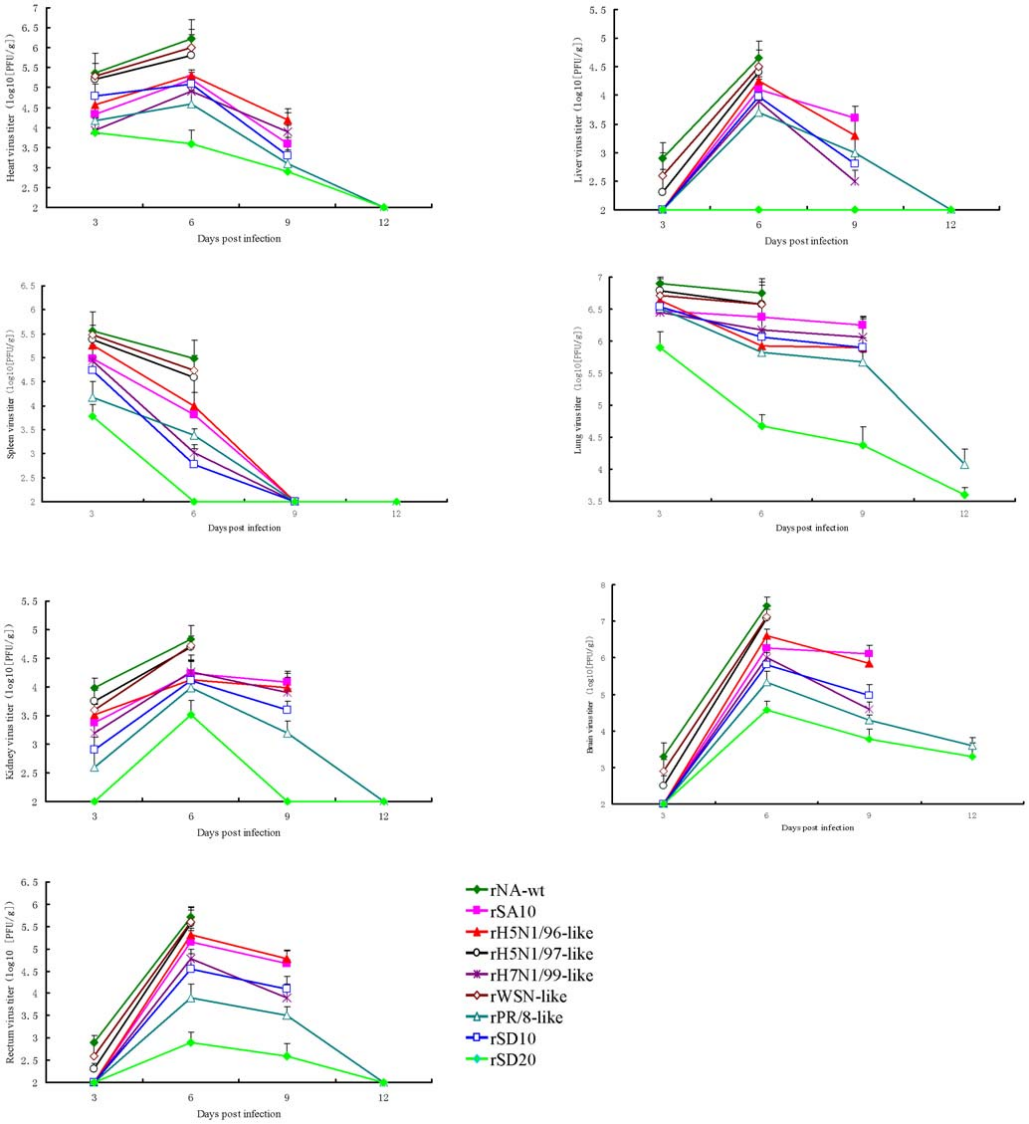
The virus distribution in different tissues of infected mice. Mice were infected with an inoculating dose of 1000EID_50_ of the recombinant viruses. On days 3, 6, 9 and 12 p.i., three mice from each group were sacrificed and heart, liver, spleen, lung, kidney, brain and rectum samples were harvested. Virus titers in the tissue homogenates were determined by plaque assay on MDCK cells. The virus titer in the tissues was expressed in log10 pfu/g. The lower limit of virus detection was 2 log10 PFU per 1 g of tissue.

The distribution of the recombinant viruses in mouse tissue was reported in Fig. 5. In mice infected with the recombinant viruses, we recovered the virus from a variety of organs, including heart, liver, spleen, lung, kidney, brain and rectum. As shown in Fig. 5, the rH7N1/99-like, rH5N1/96-like, rPR/8-like, rSA10 and rSD10 showed lower tissue virus titer than did rNA-wt (*P*<0.05). The rSD20 showed significantly lower tissue virus titer than did rNA-wt (*P*<0.001). The rNA-wt replicated to the highest titers in all tissues compared with the other recombinant viruses, which indicated rWSN-like, rH5N1/97-like, rH7N1/99-like, rH5N1/96-like, rPR/8-like, rSA10, rSD10 and rSD20 displayed an attenuated phenotype. The stalkless mutant rSD20 displayed a high attenuation to mice and caused the lowest replication in mouse tissues.

## Discussion

By using the PB2, PB1, PA, NP, M and NS from A/WSN/33 and the HA and NA of a highly pathogenic avian isolated strain, A/chicken/Hubei/327/2004/H5N1, as genetic backbone, we constructed nine H5N1 recombinant viruses that shared seven genes and differed from each other only in NA gene. Under natural conditions, the reassortment of the segmented viral genes and the generation of a reassortant H5N1 virus are common. So study on the recombinant H5N1 virus with different derived gene may better clarify the role of NA with different stalk-motif in the natural reassortment of H5N1 virus.

The virulence and pathogenesis of influenza virus strains is a multigenic trait and has been shown to be caused by the specific sequence of viral proteins including the external surface glycoproteins, HA and NA [15]–[17], the three internal polymerase proteins [16]–[19], and the two nonstructural proteins PB1-F2 20, 21 and NS1 [22], [23]. HA binds to sialic acid-containing receptors on target cells to initiate virus infection, whereas NA activity cleaves sialic acids from cellular receptors and promotes the release of virus from infected cells and prevents HA-mediated self-aggregation by desialylation of viral and cellular glycoconjugates [24], [25]. The functional balance between HA receptor affinity and NA sialidase activity is necessary for efficient influenza virus replication and HA and NA activities in influenza viruses need to be highly balanced in order to allow productive influenza virus infection or be well adapted to their hosts [26], [27]. Study has shown the interdependence of the HA glycosylation and NA stalk length [28]. Most H5N1 influenza viruses with a 20-amino acid deletion in the 49^th^ to 68^th^ in the stalk region contain glycosylation at aa170 (or aa169), but the H5N1 influenza viruses with entire stalk region lack the glycosylation. Glycosylation of the HA can lead to a reduced receptor affinity of the HA [29]. The glycosylation at aa170 flanking the receptor binding site of HA might decrease the binding affinity for sialic acid and the deficiency in NA activity conferred by the shortened protein stalk could be compensated by a decreased receptor binding affinity of the HA that restores the functional balance of HA and NA.

In this study, the recombinant virus with NA-wt stalk-motif displayed the highest virulence and pathogenesis, while the other recombinant viruses with WSN-like, H5N1/97-like, H7N1/99-like, H5N1/96-like, PR/8-like, SA10, SD10 or SD20 stalk-motif displayed an attenuated phenotype. This indicates that the NA stalk-motif is associated with virulence and pathogenesis of H5N1 influenza virus.

For the segmented nature of the influenza genome, the generation of a reassortant virus is an important mechanism of the variation of influenza virus. Theoretically, each of these NAs of N1 subtype with different stalk-motifs may be one genetic donor in the reassortment of H5N1 influenza A virus. In this study, the recombinant H5N1 virus with H7N1/99-like, H5N1/96-like or PR/8-like stalk-motif displayed a significantly attenuated phenotype compared with rNA-wt, which might lead to that H7N1/99-like, H5N1/96-like or PR/8-like stalk-motif was not able to be the predominant phenotype and circulate popularly in the evolution of H5N1 influenza virus. In the H5N1 isolates from 2000 to 2007, H7N1/99-like or PR/8-like stalk-motif was not observed, and H5N1/96-like stalk-motif gradually disappeared and was not observed in recent H5N1 isolates.

All the recombinant viruses with longer (H5N1/96-like and SA10) or shorter stalk-motif (SD10 and SD20) displayed an attenuated phenotype compared with rNA-wt. Castrucci et al. indicated that the stalkless mutant H1N1 virus did not grow in eggs and could not cause systemic infection in mice and had lost its neurovirulence [3]. In our study, the stalkless H5N1 virus still replicated, but to a titer slightly lower than that of the rNA-wt in eggs, and did not cause any disease signs or deaths in chicken (IVPI = 0). The stalkless H5N1 virus could also cause systemic infection in mice similar to that of the rNA-wt and did not lose its neurovirulence, and the virus could be recovered from the brain, which might be the particularity of H5N1 influenza virus differed from H1N1 influenza virus.

The replacement of the NA-wt stalk-motif by WSN-like or H5N1/97-like stalk-motif did not significantly influence biologic characteristic and pathogenesis of the recombinant H5N1 virus. The analysis of all NA sequences of H5N1 subtype from 2000 to 2007 in influenza virus resource databases at http://www.ncbi.nlm.nih.gov/ showed that the H5N1/97-like stalk-motif was observed in three H5N1 strains, A/duck/Vietnam/1/2005, A/goose/Vietnam/3/05 and A/duck/Vietnam/8/05, and all the three viruses were highly lethal and killed chickens within 30 hours post inoculation, with the IVPI ranging from 2.7 to 2.9 [30]. However, the WSN-like or H5N1/97-like stalk-motif was not predominant phenotype in H5N1 isolates after 2000.

From 2000, a 20-amino acid deletion in the 49^th^ to 68^th^ in the stalk region was observed in H5N1 influenza virus, which was different from previous H5N1 isolates from 1996 to 1999. This showed a change in the number and position of amino acid deletion in NA, which provided couple lines of evidence for a separate NA gene introduction, leading to the emergence of new H5N1 virus genotype after the year 2000. It was speculated that the deletion in the NA stalk may be associated with adaptation of influenza viruses to land-based poultry and increased virulence and pathogenesis in poultry and mammalian [31], [32]. In this study, the virus with the special NA stalk-motif (NA-wt) possessed the highest virulence and pathogenicity in chicken and mice. The percentage of H5N1 isolates with the special NA stalk-motif gradually increased from 2000 (15.8%) to 2007 (100%). Significantly, the special NA stalk-motif was observed in 1 of the total 5 H5N1 human influenza virus strains in 2003 and in all 173 strains isolated from 2004 to 2007, which may be associated with the gradual transmission of H5N1 influenza virus to human.

The gradually increasing emergence of the special NA stalk-motif in H5N1 influenza virus deserves attention by all. It is clear that the NA stalk-motif plays a critical role in virulence and pathogenesis of H5N1 avian influenza virus and the special NA stalk-motif (a 20-amino acid deletion in the 49^th^ to 68^th^ in the stalk region) may be an important one among the reasons contributing to the emergence of increased virulence H5N1 isolated strain since 2000.
